# The Middlesex Hospital Appeal

**Published:** 1910-10-01

**Authors:** 


### Editor's Letter-Box.

### THE MIDDLESEX HOSPITAL APPEAL.

*To the Editor of* The Hospital.

Sir,—I am happy to be able to inform you that I have now received the £20,000 I asked for to enable me to remove the debt from the Middlesex Hospital, a generous friend, who desires to remain anonymous, having just handed me a cheque for £231 in order to complete that sum.

I find some difficulty in suitably expressing the deep sense of gratitude I feel towards all those who have so loyally responded to my appeal on behalf of an institution in whose activities they have now shown, by their practical sympathy, the highest confidence and appreciation.

Rich and poor alike have contributed to the success of my effort, for the sums I have received range from one thousand guineas to threepence. It has afforded me the greatest gratification to observe the generosity of those who owe their present freedom from disease or relief from pain to the hospital's kindly influence, and I venture to say that no stronger proof could be found of the value of this ancient charity than that those who were once under its care should have come forward, cheerfully and often with much self-sacrifice, to share its burden in its hour of need.

To each and every contributor I once again offer my sincerest thanks, and I also take this opportunity gratefully to acknowledge my indebtedness to the Press for the valuable assistance they have afforded me by bringing the needs of the hospital prominently before their readers.

But my task is not yet finished. The debt of £20,000 has, it is true, been removed, but that liability represented the accumulated deficits between income and expenditure for three years, and from this it is obvious that, until a steady and permanent addition of £7,000 per annum is made to the hospital's income, its financial position is not secure, and every third year the Governors will find themselves face to face with a crisis similar to that which has now happily been averted.

It is my ambition to substitute, for such a hand-to-mouth administration as this, one which will provide the Governors with an income sufficient to meet the normal expenses of the year, so that they may apply themselves solely to seeing that it is expended to the best advantage in the interests of those whom the hospital serves, and, directly I am able to do so, it is my intention to devote my time and energy to building up an adequate annual subscription and donation list. I feel sure that my confidence in the generosity of those to whom I apply will again be fully justified.

Yours truly,

Francis of Teck.

Chairman of the Weekly Board of Governors.

[We refer to this letter in "The Week's Work."—Ed. The Hospital.]

